# Circ_0003570 Suppresses the progression of hepatocellular carcinoma through miR-182-5p/STARD13 regulatory axis

**DOI:** 10.1186/s12575-022-00176-w

**Published:** 2022-10-14

**Authors:** Xu Zhang, Wenwen Chen, Dan Guo, Yarui Li, Yan Zhao, Mudan Ren, Guifang Lu, Xinlan Lu, Shuixiang He

**Affiliations:** Department of Gastroenterology, the First Affiliated Hospital of Xi’an Jiaotong University, Xi’an, Shaanxi 710061 People’s Republic of China

**Keywords:** Hepatocellular carcinoma, circ_0003570, miR-182-5p, STARD13

## Abstract

**Background:**

Emerging evidence have revealed that circRNAs exert important biological effects in the development and progression of various diseases, including cancer. Our study aimed to elaborated the biological effects of hsa-circ_0003570 in hepatocellular carcinoma (HCC) development at the molecular level.

**Results:**

The results of functional experiments showed that knockdown of circ_0003570 induced HCC cell growth, migration and invasion, whereas overexpression of circ_0003570 presented the opposite effects. In vivo experiments, xenograft tumors grown from circ-overexpressed cells had smaller tumor volume and weight than the control group. Further investigations suggested that circ_0003570 may function as a competing endogenous RNA via competitively binding miR-182-5p and thereby regulating the repression of downstream target gene STARD13, which were demonstrated by dual luciferase reporter assay and functional rescued experiments.

**Conclusions:**

Taken together, circ_0003570 suppresses the development of HCC by modulating miR-182-5p/STARD13 axis.

**Supplementary Information:**

The online version contains supplementary material available at 10.1186/s12575-022-00176-w.

## Introduction

Hepatocellular carcinoma (HCC) is the sixth most common cancer and the third leading cause of cancer death around the world. Every year, approximately 840,000 new cases are diagnosed with HCC and at least 780,000 patients die of this kind of cancer [1, 2]. Currently, some therapeutic strategies for HCC, including hepatectomy, radiofrequency ablation (RFA), hepatic transarterial chemoembolization (TACE), chemotherapy, and molecular targeted therapy, have yielded great progress. However, these treatments are not effective for advanced HCC owing to the high recurrence rate and metastatic rate [3]. Therefore, there is an urgent need to discover new targets and better understand the molecular mechanisms that regulating HCC progression, with the aim of opening up new approaches of diagnostic and therapeutic strategies and improving the prognosis of HCC patients.

As a novel class of non-coding RNAs, circular RNAs (circRNAs) exhibit covalently closed loop structures with neither 5’cap nor 3’ polyadenylated tail [2]. An increasing number of studies have showed that circRNAs exert important biological effects by mediating gene transcription, modulating protein translation, binding RBPs, sponging miRNAs, indicating that they are involved in the development and progression of various diseases, including cancer [4–6]. Recently, the most frequently investigated function of circRNAs is its role as “miRNA sponges” to mediate target gene expression since some have binding sites with miRNAs [7]. For instance, circ_0001955 facilitates proliferation, migration, and invasion of HCC through sponging miR-145-5p [8]. Circ_0001955 enhances HCC tumorigenesis by competitively bound to miR-516a-5p to release TRAF6 and MAPK11 [9]. Exosome-transmitted circular RNA circ_0051443 suppresses HCC progression by miR-331-3p/BAK1 axis [10]. More recently, some bioinformatic analyses have identified various circRNAs that differentially expressed in HCC tissues and cell lines, and they may participate in tumor progression [11–13]. Although circRNAs are considered to have a significant effect on HCC development and progression, the exact mechanisms remain largely unclear.

In the current study, we characterized a novel circRNA originated from FAM53B gene (hsa_circ_0003570 in circBase: http://www.circbase.org). Circ_0003570 is located on chr 10: 126,370,175–126,384,781, containing a spliced sequence length of 828 bp. A clinicopathological study has found that circ_0003570 was differentially expressed in HCC tissues and cell lines, and its expression level might be correlated with patient prognosis [14]. However, the functions and molecular regulatory mechanisms of circ_0003570 have not been studied yet. First, our study found that circ_0003570 inhibited the proliferation and metastasis of HCC in vitro and in vivo. Furthermore, we were pleasantly surprised to find that circ_0003570 modulated proliferation, migration and invasion of HCC cells by binding miR‐182‐5p as a miRNA sponge to regulate STARD13 expression. Therefore, we propose that the circ_0003570/miR‐182‐5p/STARD13 axis may play an important role in hepatocellular carcinoma progression.

## Methods

### Cell culture

All HCC cells including Huh7, Hep3B, HepG2, MHCC-97L, SMCC-7721, and immortalized human hepatic cell LO2 were obtained from the Type Culture Collection of the Chinese Academy of Sciences. All cells were cultured in Dulbecco’s modified Eagle’s medium (DMEM)/high glucose(Gibco, USA), supplement 10% FBS (Gibco, USA) and penicillin–streptomycin (100 U/mL and 100 ug/mL, respectively) and maintained at 37˚C in 5% CO2 humidified incubator.

### Oligonuleotide transfection

Small interfering RNA (siRNA), miRNA mimics, miRNA inhibitors and negative control oligos were synthesized by GenePharma (Shanghai, China). The sequences.

are listed in Table 1. Transfection was performed using Lipo8000 (Biyotime, China).

**Table 1:** The sequences of siRNAs, miRNA mimics, miRNA inhibitors and negative controls

	sense(5'-3')	antisense(5'-3')
si-has-circ_0003570	CUUGCCAAGAUGGCACAGCTT	GCUGUGCCAUCUUGGCAAGTT
miR-182-5p mimics	UUUGGCAAUGGUAGAACUCACACU	UGUGAGUUCUACCAUUGCCAAAUU
miR-182-5P inhibitor	AGUGUGAGUUCUACCAUUGCCAAA	
mimics control	UUGUACUACACAAAAGUACUG	
inhibitor control	CAGUACUUUUGUGUAGUACAA	
siRNA Negative Control	UUCUCCGAACGUGUCACGUTT	ACGUGACACGUUCGGAGAATT

### Plasmid construction and stable transfection

The circ_0003570 overexpression plasmids and lentiviruses were synthesized by (Hanheng Biotechnology Co., Ltd., Shanghai, China). The constructs were confirmed by sequencing. Afterwards, HepG2 and MHCC-97L cells were infected with a circ_0003570 overexpression lentivirus or a negative control lentivitus. For stable cell transfection, the transfected cells were selected with puromycin for 2 weeks. Finally, the transfection efficiency was examined via qRT‐PCR analysis.

### The isolation of RNA and quantitative real‐time PCR (qRT‐PCR)

Total RNA from cells was extracted with Trizol reagent (Invitrogen, Carlsbad, CA) according to the manufacturer’s protocol. Then, the cDNA was synthesized following the protocol of *Evo M-MLV* RT Premix for qPCR (Accurate Biotechnology, Hunan, China) and Goldenstar™ RT6 cDNA Synthesis Kit (TsingKe, China). Quantitative real-time PCR was carried out using Master qPCR Mix (TsingKe, China) on a CFX96 Real-Time PCR system (Bio-Rad, CA, USA). The relative gene expression was calculated using the 2^−ΔΔCt^ method. β-actin was served as the internal reference of mRNA/circRNA, and U6 was considered as the internal reference of miRNA. The primers in the study were showed in Table 2.

**Table 2:** The primers in the study

	Forward Primer	Reverse Primer	common reverse primer
has-circ_0003570	GCACAGCACACGCCAAA	AAGAGGGCATTTCCTGTCCA	
miR-182-5p	CGCTTTGGCAATGGTAGAACT	GTCGTATCGACTGCAGGGTCCGAGG TATTCGCAGTCGATACGACAGTGTG	ACTGCAGGGTCCGAG GTATT
STARD13	CGAGGAGACAGAAATGGGTCA	TCCACTGCTTTCGCTGTGAAT	
β-actin	ATCGTGCGTGACATTAAGGAGAAG	AGGAAGGAAGGCTGGAAGAGTG	
U6	GGAACGATACAGAGAAGATTAGC	TGGAACGCTTCACGAATTTGCG	

### Colony formation assay

After 24 h of transfection, cells were planted into 6‐well plate with 1000 cells per well. After about 1 weeks, when clones formed by single cell possessed at least 50 cells, the clones were fixed with methanol for 10 min and next 0.1% crystal violet were used for staining about 10 min. Finally, the clones were counted for statistical analysis after washed and aired.

### MTT assay

Cell viability was assessed by MTT (3‐(4, 5‐dimethylthiazol‐2‐yl)‐2,5‐diphenyltetrazolium bromide) assay. Cells were seeded into 96‐well plates at a density of 5000 cells per well. 24 h after seeding, transfection was conducted as the reagent's protocol. After transfection for 24 h, 48 h, 72 h, 96 h, 10 μL of 5 mg/mL MTT was added into the medium in the dark and then cultured for 4 h in incubator. The suspension was then discarded and 150 μL DMSO was added to dissolve the crystal. Using an ELISA reader (Bio-Rad, Hercules, CA, USA), cells viability were measured by the optical density (OD) at 490 nm. Triplicate experiments were performed for each assay.

### Transwell assay

After 48 h of cell transfection, 5 × 10^4^ cells were respectively seeded into 8 μm pore size transwell 24‐well chambers (Merck Millipore) coated with Matrigel (BD Biosciences) for invasion assay and uncoated chambers for migration. On the top of chamber is serum-free DMEM medium and lower wells were supplemented 10% FBS. After incubation for further 24 h, chambers were taken out and washed off the non‐invaded and non‐migrated cells gently on the upper membrane. Then the chambers were fixed with 95% ethyl alcohol for 10 min and stained by crystal violet for 10 min, and washed in PBS. Stained cells were observed under optical microscope and calculated.

### Wound healing assay

After cell transfection, the HCC cells were cultured for 24 h to achieve 90% cell density in six-well plates, and the wells were previously externally marked with a straight black line on the bottom to use as guides for the subsequent photography. Wound (perpendicular to the guide lines) was scratched by a 200 µl sterile tip, and then washed off the floating cells. Under the microscope, the wound size was measured and photographed at 0, 24, and 48 h. All pictures were collected under microscope. Images were aligned using the orientation line to ensure that the identical spots were followed over time.

### Dual‐luciferase reporter assay

Bioinformatics tools were used to analyze the miR-182-5p binding sites on circ_0003570 and STARD13 binding sites on miR-182-5p. Cells were seeded into 96‐well plate at approximately 60%‐80% confluence before transfection. Plasmid vector including the wild-type (WT) or mutant (Mut) 3′UTR of circ_0003570 or STARD13 (Hanheng Biotechnology, China) together with miR-182-5p mimics/inhibitor or NC (GenePharma) were co-transfected using the Lipo8000 reagent (Biyotime, China). The basic vector used for the reporter plasmid was psiCheck2 plasmid. The luciferase activity was examined using the Dual Luciferase Assay Kit (Biyotime, China). The Renilla luciferase activity was considered as control.The sequence of synthesized reporter plasmids (wt-circ_0003570-luc, mut-circ_0003570-luc; wt-STARD13-luc, mut-STARD13-luc) was showed in Table 3. The vector maps of wt-circ_0003570-luc, mut-circ_0003570-luc, wt-STARD13-luc, and mut-STARD13-luc used in this study were presented in Fig. S1.

**Table 3:** The sequence of luciferase reporter plasmids

	sequence
PC-circ_0003570-WT	**CACACGCCAAAGAAGATGAGTCAAGGACCTACACTTTTCTCTTGTGGAATTATGGAAAATGACAGATGGCGAGACCTGGACAGGAAATGCCCTCTTCAGATTGACCAACCGAGCACCAGCATCTGGGAATGCCTGCCTGAAAAGGACAGCTCACTATGGCACCGGGAGGCAGTGACCGCCTGCGCTGTGACCAGTCTGATCAAAGACCTCAGCATCAGCGACCACAACGGGAACCCCTCAGCACCCCCTAGCAAGCGCCAGTGCCGCTCACTGTCCTTCTCCGATGAGATGTCCAGTTGCCGGACATCATGGAGGCCCTTGGGCTCCAAAGTCTGGACTCCCGTGGAAAAGAGACGCTGCTACAGCGGGGGCAGCGTCCAGCGCTATTCCAACGGCTTCAGCACCATGCAGAGGAGTTCCAGCTTCAGCCTCCCTTCCCGGGCCAACGTGCTCTCCTCACCCTGCGACCAGGCAGGACTCCACCACCGATTTGGAGGGCAGCCCTGCCAAGGGGTGCCAGGCTCAGCCCCGTGTGGACAGGCAGGTGACACCTGGAGCCCTGACCTGCACCCCGTGGGAGGAGGCCGGCTGGACCTGCAGCGGTCCCTCTCTTGCTCACATGAGCAGTTTTCCTTTGTGGAATACTGTCCTCCCTCAGCCAACAGCACACCTGCCTCAACACCAGAGCTGGCGAGACGCTCCAGCGGCCTTTCCCGCAGCCGCTCCCAGCCGTGTGTCCTTAACGACAAGAAGGTCGGTGTTAAAAGGCGGCGCCCTGAAGAAGTGCAAGAGCAGAGGCCTTCTCTAGACCTTGCCAAGATGGCACAG**
PC-circ_0003570-Mut	**CACACGCCAAAGAAGATGAGTCAAGGACCTACACTTTTCTCTTGTGGAATTATGGAAAATGACAGATGGCGAGACCTGGACAGGAAATGCCCTCTTCAGATTGACCAACCGAGCACCAGCATCTGGGAATGCCTGCCTGAAAAGGACAGCTCACTATGGCACCGGGAGGCAGTGACCGCCTGCGCTGTGACCAGTCTGATCAAAGACCTCAGCATCAGCGACCACAACGGGAACCCCTCAGCACCCCCTAGCAAGCGCCAGTGCCGCTCACTGTCCTTCTCCGATGAGATGTCCAGTTGCCGGACATCATGGAGGCCCTTGGGCTCCAAAGTCTGGACTCCCGTGGAAAAGAGACGCTGCTACAGCGGGGGCAGCGTCCAGCGCTATTCCAACGGCTTCAGCACCATGCAGAGGAGTTCCAGCTTCAGCCTCCCTTCCCGGGCCAACGTGCTCTCCTCACCCTGCGACCAGGCAGGACTCCACCACCGATTTGGAGGGCAGCCCTGCCAAGGGGTGCCAGGCTCAGCCCCGTGTGGACAGGCAGGTGACACCTGGAGCCCTGACCTGCACCCCGTGGGAGGAGGCCGGCTGGACCTGCAGCGGTCCCTCTCTTGCTCACATGAGCAGTTTTCCTTTGTGGAATACTGTCCTCCCTCAGCCAACAGCACACCTGCCTCAACACCAGAGCTGGCGAGACGCTCCAGCGGCCTTTCCCGCAGCCGCTCCCAGCCGTGTGTCCTTAACGACAAGAAGGTCGGTGTTAAAAGGCGGCGCCCTGAAGAAGTGCAAGAGCAGAGGCCTTCACGAGAACGTCCGATGATGGCACAG**
PC-STARD13-WT	**cagtgcatctttttaaaacaaggaaaagggaaaaaaaggagaaaaaacgatgcatcaagcttgtttgtcaaataccacagtattttattcattgttatcttgccaatggaaataaagtatgatattgcatttaaatattatatttatacctcatgtatatttttacctcaattgttgatatcaatcatcaattgtaaata**
PC-STARD13-Mut	**cagtgcatctttttaaaacaaggaaaagggaaaaaaaggagaaaaaacgatgcatcaagcttgtttgtcaaataccacagtattttattcattgttatcAtCcGaTtggaaataaagtatgatattgcatttaaatattatatttatacctcatgtatatttttacctcaattgttgatatcaatcatcaattgtaaata**

### Western blot analysis

After transfection for 72 h, all proteins were isolated by RIPA (Pierce). Proteases and phosphatases were added to RIPA in advance to prevent protein degradation. Protein samples were loaded for electrophoresis (5% gel for concentration and 10% for separation), and then the proteins were transferred on a 0.45 μm or 0.22 μm pore size PVDF membrane (Merck Millipore). After blocking with 5% defatted milk gently for 1 h, the membrane was incubated at 4 °C overnight with primary antibodies (STARD13; Proteintech) at 1:2000 dilution. The next day, the membrane was washed extensively and incubated with secondary antibodies (at 1:2500 dilution; Zhuangzhi Biology, China) for 1 h at room temperature. Proteins bands were detected by using ECL immunoblotting kit (Millipore, USA) following the manufacturer’s protocol.

### Tumorigenicity assay

Male BALB/c nude mice (4-week-old) were got from the animal laboratory center of Xi’an Jiao Tong University. The mice were divided into two groups randomly, respectively accepted subcutaneously injection for 100μL of stably expressed circ_0003570 or control HepG2 cells in PBS, which contained approximately 5 × 10^6^ cells. After one month or more, all mice involved were sacrificed. 100μL of 2 × 10^6^ lentivirus-transduced HepG2 cells were injected from tail vein for metastasis and after 2 months mice were sacrificed and lung specimens were collected for research next. The study was conducted in accordance with the National Institutes of Health “Guide for the Care and Use of Laboratory Animals” and approved by the medical ethics committee of Xi’an Jiao Tong University.

### H&E staining and Immunohistochemistry (IHC)

All tissues were fixed with 10% of formalin for 48 h and embedded with paraffin wax, which were cut for 4 mm thick. After dewaxed by xylene and hydrated using ethyl alcohol of step-rising concentration. Then, H&E staining was preformed and Sections were observed using microscope. For IHC, primary antibody of STARD13 (Proteintech) at dilution rate 1:200 were incubated overnight at 4 °C. The next day, secondary antibodies were incubated at room temperature, and then, slides were stained with DAB and hematoxylin. Images were collected under microscope after dehydration and transparency.

### Statistical analysis

All data were showed as means ± SEM and analyzed by SPSS 23.0 (IBM, SPSS, Chicago, IL, USA) and GraphPad Prism 6.0 (GraphPad Software, CA, USA). Student’s t-test or one-way ANOVA was performed to compare the difference between the groups, and Pearson’s correlation coefficient analysis was applied to analyze the correlation between two groups. A two-tailed P < 0.05 was regarded as statistically significant, and *P* < 0.01 was very significant.

## Results

### Knockdown of circ_0003570 promotes proliferation, migration and invasion of HCC cells in vitro

**Fig. 1:**
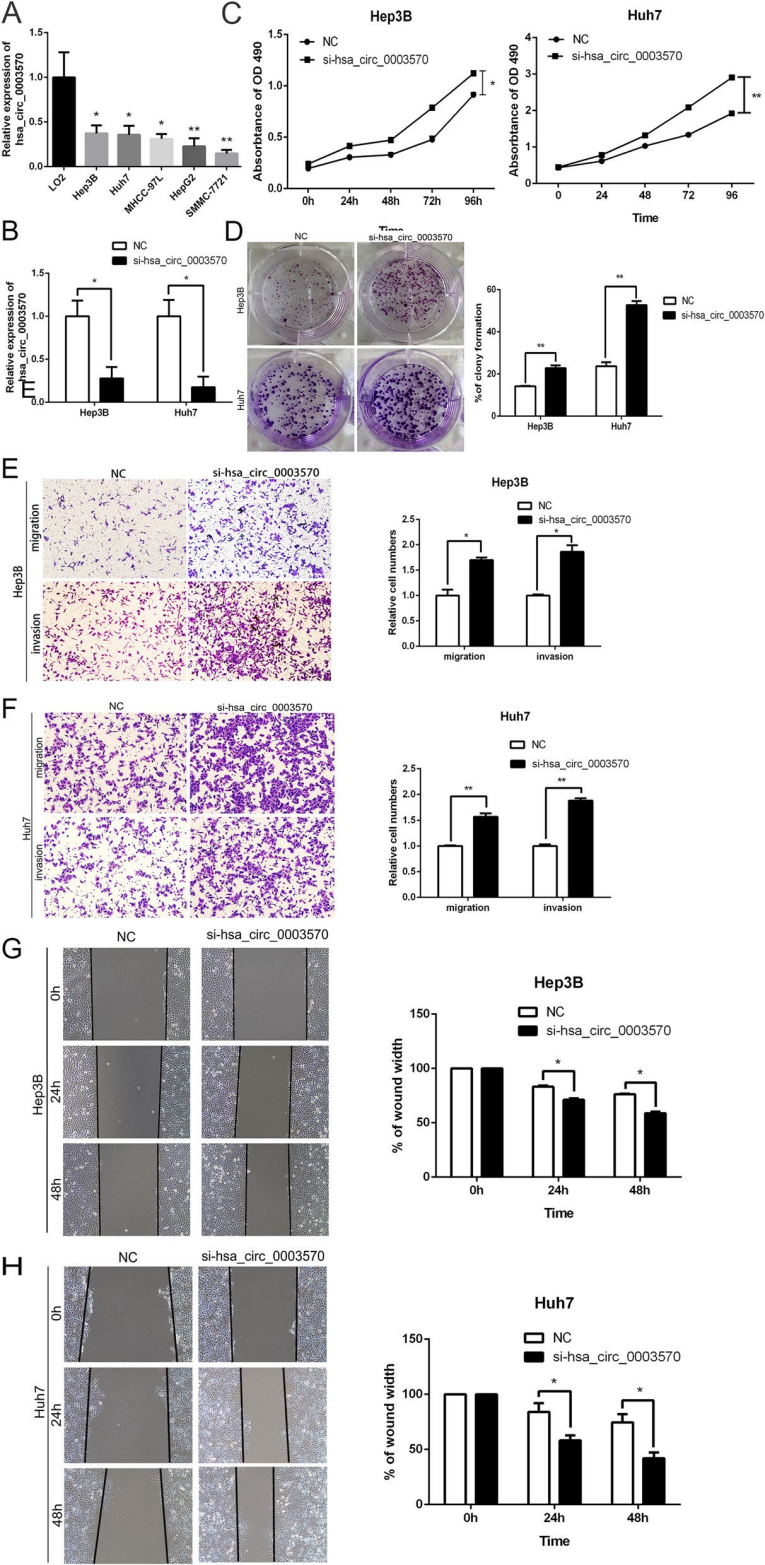
Knockdown of circ_0003570 promotes proliferation, migration and invasion of HCC cells in vitro. **A** qRT-PCR analysis of circ_0003570 expression in HCC cell lines and the immortalized human hepatic cell line LO2, *P < 0.05; **P < 0.01. **B** qRT-PCR analysis of circ_0003570 expression following transfected HCC cells with the siRNA of circ_0003570, *P < 0.05. **C** MTT assays and **D** colony formation assays showing that downregulation of circ_0003570 promoted the proliferation of HCC cells, *P < 0.05. **E**, **F** Transwell assays showed circ_0003570 knockdown reduced the migration and invasion of HCC cells. *P < 0.05. **G**, **H** Representative images of wound healing assays after circ_0003570 silencing. Student’s t-test was performed to compare the difference between two groups. *P < 0.05

Firstly, we investigated the expression of circ_0003570 in HCC cells, including Huh7, Hep3B, MHCC‐97L, HepG2, SMMC‐7721 and immortalized human hepatic cell LO2 by qRT‐PCR. As presented in Fig. 1A, the expression levels of circ_0003570 in the human HCC cell lines were markedly lower compared with LO2 cell line, among which Huh7 cell line and Hep3B cell line harbored the highest circ_0003570 expression. In order to further investigate the biological function of circ_0003570 in vitro, its specific siRNA was utilized to knock down circ_0003570 in Hep3B, Huh7, and MHCC-97L cells. Here, the knockdown efficiency against circ_0003570 by siRNA was confirmed through qRT-PCR analysis (Fig. 1B). Growth curves performed by MTT assay indicated that inhibition of circ_0003570 significantly promoted Hep3B and Huh7 cell proliferation (Fig. 1C). Similar to the results of MTT assay, the results of colony formation assay showed that circ_0003570 knockdown promoted cell colony formation capabilities, when compared with negative control (Fig. 1D). Simultaneously, wound healing and transwell assays were preformed to evaluate the effects of circ_0003570 on migration and invasion of Hep3B and Huh7 cells. Transwell migration and invasion assays revealed that the cell migration and invasion capabilities of Hep3B and Huh7 cells were significantly elevated by downregulation of circ_0003570 (Fig. 1E-F). Consistent with the results of transwell assays, wound healing assay demonstrated that knockdown of circ_0003570 remarkably enhanced cell migration, as compared with si-NC group (Fig. 1G-H). Similarly results were also showed in MHCC-97L cells (Fig. S2). The flow cytometric analysis (FACS) revealed the impact of circ_0003570 knockdown on cell apoptosis, and cycle was subtle, as shown in Fig. S3.Taken together, these data indicated that circ_0003570 played a tumor-suppressor role via suppressing proliferation, migration and invasion of HCC cells in vitro.

### Overexpression of circ_0003570 suppresses proliferation, migration and invasion of HCC cells in vitro

**Fig. 2:**
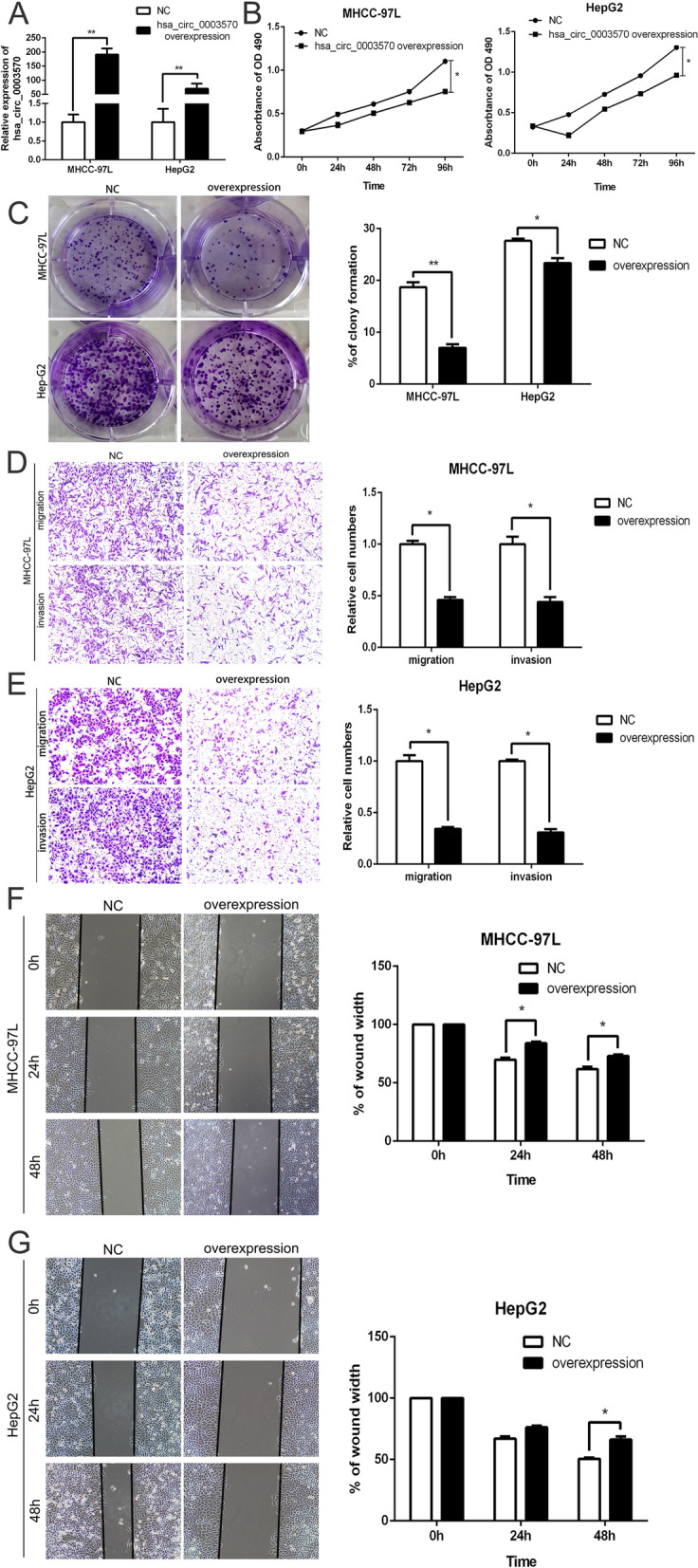
Overexpression of circ_0003570 suppresses proliferation, migration and invasion of HCC cells in vitro. **A** qRT-PCR analysis of circ_0003570 expression following transfected HCC cells with the overexpressed lentiviral vector of circ_0003570, *P < 0.05; **P < 0.01. **B** MTT assays and **C** colony formation assays showing that overexpression of circ_0003570 inhibited the proliferation of HCC cells, *P < 0.05. **D**, **E** Transwell assays showed circ_0003570 knockdown reduced the migration and invasion of HCC cells. *P < 0.05. **F**, **G** Representative images of wound healing assays after circ_0003570 silencing. Student’s t-test was performed to compare the difference between two groups.*P < 0.05

We further detected the biological function of circ_0003570 via transfecting circ_0003570 overexpression vector into HepG2 and MHCC‐97L cell lines, which have lower expression of circ_0003570. Circ_0003570 expression in HepG2 and MHCC‐97L cells strikingly enhanced after transfection with the LV-circ_0003570 in comparison with the empty vector (Fig. 2A). MTT assay presented that circ-overexpressed HCC cells exhibited a striking decline in the growth curves, compared with the control group (Fig. 2B). Consistently, colony formation assay revealed that overexpression of circ_0003570 significantly hindered colony formation capacity (Fig. 2C). Furthermore, wound healing and transwell assays showed, circ_0003570 overexpression significantly raised invasion and migration capacity of HepG2 and MHCC‐97L cells (Fig. 2D-F). The flow cytometric analysis (FACS) revealed the impact of circ_0003570 overexpression on cell apoptosis, and cycle was not significant, as shown in Fig.S3. In a word, circ_0003570 functions as a tumor suppressor in HCC progression.

### Circ_0003570 acts as a sponge of miR-182-5p to regulate STARD13

**Fig. 3:**
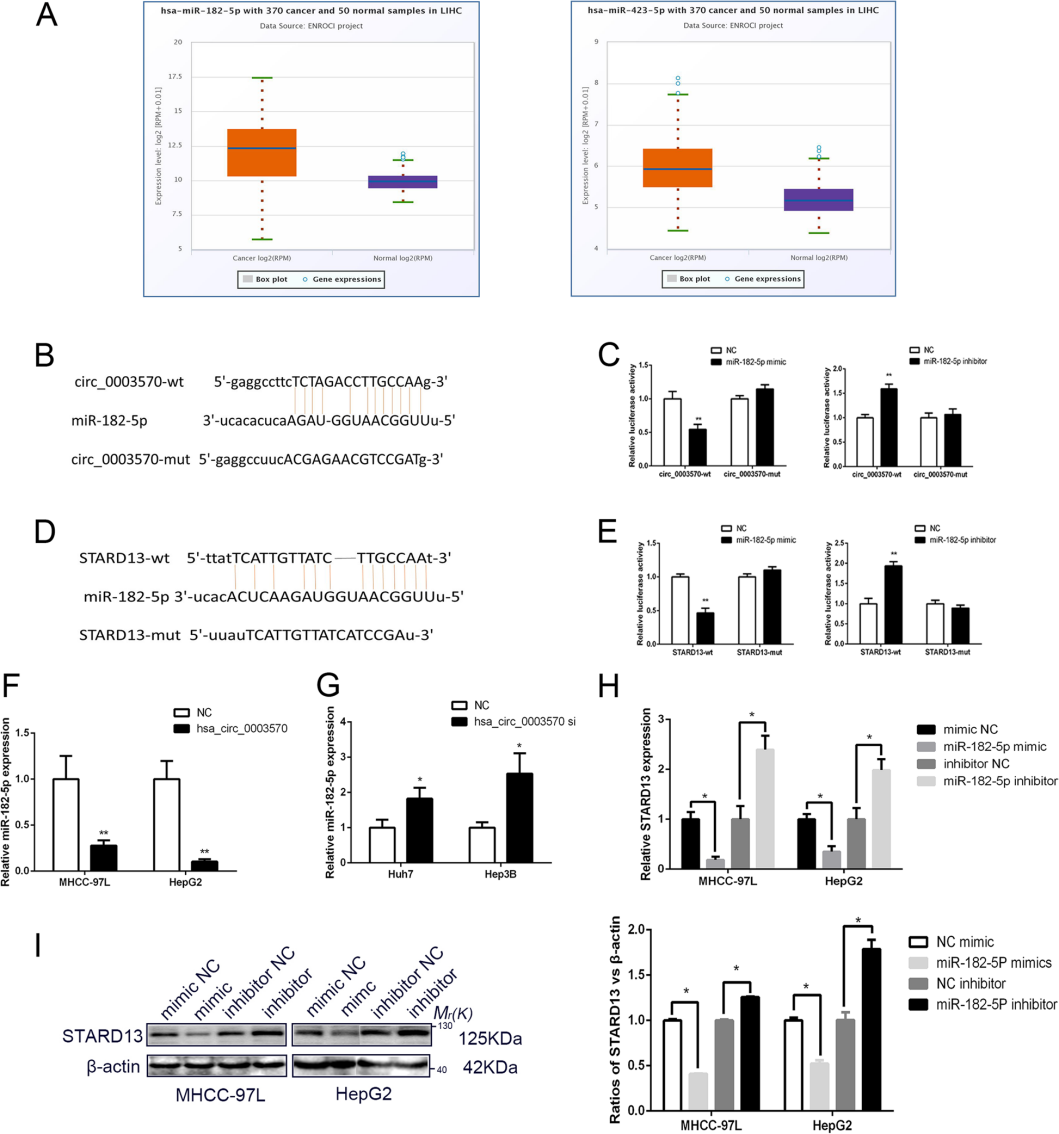
Circ_0003570 acts as a sponge of miR-182-5p to regulate STARD13. **A** Bioinformatics analysis showed the binding site between circ_0003570 and miR-182-5p. **B** A dual luciferase reporter assay was applied to verify the targeted binding effect between circ_0003570 and miR-182-5p. **P < 0.01. **C** Bioinformatics analysis showed the binding site between miR-182-5p and STARD13 3′‐UTR. **D** A dual luciferase reporter assay was applied to verify the targeted binding effect between circ_0003570 and miR-182-5p. **P < 0.01. **E**, **F** qRT-PCR analysis of miR-182-5p expression following transfected HCC cells with siRNA or overexpressed lentiviral vector of circ_0003570. *P < 0.05; **P < 0.01. **G** The STARD13 mRNA levels after miR-182-5p overexpression or downregulation were deceted by qRT-PCR. *P < 0.05. **H** The STARD13 protein levels after miR-182-5p overexpression or downregulation were deceted by western blot. Student’s t-test was performed to compare the difference between two groups

Large studies have demonstrated that many circRNAs could act as competing ceRNAs via competitively sponging miRNAs. To further elucidate the mechanism underlying the biological function of circ_0003570 in HCC progression, we explored whether miRNAs participate in HCC tumorigenesis. We applied starBase V3.0 online platform ( https://starbase.sysu.edu.cn/) and circMir 1.0 software ( http://www.bioinf.com.cn/?page_id=10) for bioinformatic prediction. 15 potential target miRNAs (miR-518e-5p, miR-520c-5p, miR-519b-5p, miR-519c-5p, miR-518c-5p, miR-526a, miR-182-5p, miR-522-5p, miR-4739, miR-3150b-3p, miR-144-3p, miR-4784, miR-1321, miR-485-5p, miR-423-5p) of circ_0003570 were predicted by starBase and circMir. Among the 15 candidate miRNAs, only miR-182-5p and miR-423-5p was significantly higher expressed in the HCC tissue (Fig. 3A). These two miRNAs were then selected as candidate miRNAs for subsequent experiments. But when circ_0003570 was overexpressed or downregulated, only miR-182-5p expression significantly changed from control. It was found that downregulation of circ_0003570 could increase the expression of miR-182-5p and overexpression of circ_0003570 suppressed miR-182-5p expression (Fig. 3F-G). Furthermore, as shown in Fig. 3B, circ_0003570 contains a probable binding site for miR-182-5p. Co-transfection of 293 T cells with plasmids circ_0003570-WT vector and miR-182-5p mimics significantly decreased luciferase reporter activity, while knockdown of miR-182-5p raised the luciferase activity of circ_0003570. However, mutating the putative binding site of miR-182-5p (circ_0003570-Mut) contributed to compete abrogation of aforementioned effects (Fig. 3C).

Since miRNAs exert biological effects by targeting specific genes, it is essential to predict potential target genes for miRNAs [15]. Previous studies[16, 17] revealed that STARD13 could be a HCC suppressor and a potential miRNA sponge, and its related ceRNA networks might be a rational therapeutic or diagnostic chance for HCC progression. In our study, we predicted that STARD13 might be a potential candidate target gene of miR-182-5p, using starBase V3.0 ( https://starbase.sysu.edu.cn/)and TargetScan Human 7.2 software ( http://www.targetscan.org/vert_72/). Bioinformatics analysis predicted that miR-182-5p could interact with STARD13 3′-UTR (Fig. 3D). Then, luciferase reporter vectors containing STARD13 3′-UTR were constructed, and the results of luciferase reporter assay showed that co-transfection of 293 T cells with PC-STARD13-WT vector and miR-182-5p mimics significantly reduced luciferase reporter activity, whereas miR-182-5p inhibitor improved the luciferase activity. However, PC-STARD13-Mut in miR-182-5p’s putative targeting sites did not resulted in above effects (Fig. 3E). These results confirmed that miR-182-5p targets circ_0003570 and STARD13. Meanwhile, upregulation of miR-182-5p markedly suppressed STARD13 RNA levels in MHCC-97L and HepG2 cells. Conversely, these results were reversed when miR-182-5p was downregulated (Fig. 2H). Subsequently, we investigated the effect of miR-182-5p on STARD13 on protein level by Western blot. Corresponding with above results, STARD13 protein expression was sharply decreased when miR-182-5p was increased, while the expression of STARD13 was elevated in miR-182-5p inhibitor group (Fig. 3I). Taken together, we inferred that circ_0003570 decoys miR-182-5p to regulate the expression of STARD13.

### MiR‐182‐5p promotes proliferation, migration and invasion of HCC cells

In our study, we investigated the function of miR-182-5p in vitro to confirm its promotion on HCC progression. Following transfection of MHCC‐97L and HepG2 cells with scrambled miR, miR-182-5p mimics or a miR-182-5p inhibitor, the expression level and the transfection efficiency of miR-182-5p were first determined by qRT-PCR (Fig. S4A). In function experiments, MTT assay revealed that miR-182-5p mimics improved proliferative capacity of HCC cells while miR-182-5p inhibitor repressed cell proliferation (Fig. S4B-C). In addition, the results of clone formation assay demonstrated that the number of clones increased in miR-182-5p mimics group while decreased in miR-182-5p inhibitor group (Fig. S4D). Considering transwell assay, cells in miR-182-5p mimics group obviously migrated and invaded more quickly than that in miR-182-5p mimics NC group, while miR-182-5p inhibitor obviously attenuated migration and invasion capacity of HCC cells (Fig. S4E-F). Furthermore, for wound healing assay, miR-182-5p mimics evidently advocated HCC cells migration abilities while knockdown of miR-182-5p severely inhibited HCC cells mobility (Fig. S4G-H). In short, miR-182-5p accelerates proliferation and metastasis of HCC cells in vitro.

### STARD13 inhibits proliferation, migration and invasion of HCC cells

The function of STARD13 was then investigated to confirm its inhibition on HCC progression. Following transfection of HCC cells with siRNA or a STARD13 overexpression plasmid, the transfection efficiency of STARD13 was detected by western blotting (Fig. S5A). In function experiments, MTT assay revealed that STARD13 hindered proliferative capacity of HCC cells while STARD13 knockdown enhanced cell proliferation (Fig. S5B). In addition, the results of clone formation assay demonstrated that the number of clones increased in STARD13 underexpressing group while decreased in STARD13 overexpressing group (Fig. S5C). For transwell and wound healing assays, STARD13 downregulation evidently advocated HCC cells migration and invasion abilities while upregulation of STARD13 severely inhibited HCC cells mobility (Fig. S5D-E). In conclusion, STARD13 inhibits proliferation and metastasis of HCC cells in vitro.

### Circ_0003570 suppresses HCC proliferation, migration and invasion via the miR-182-5p/STARD13 axis

**Fig. 4:**
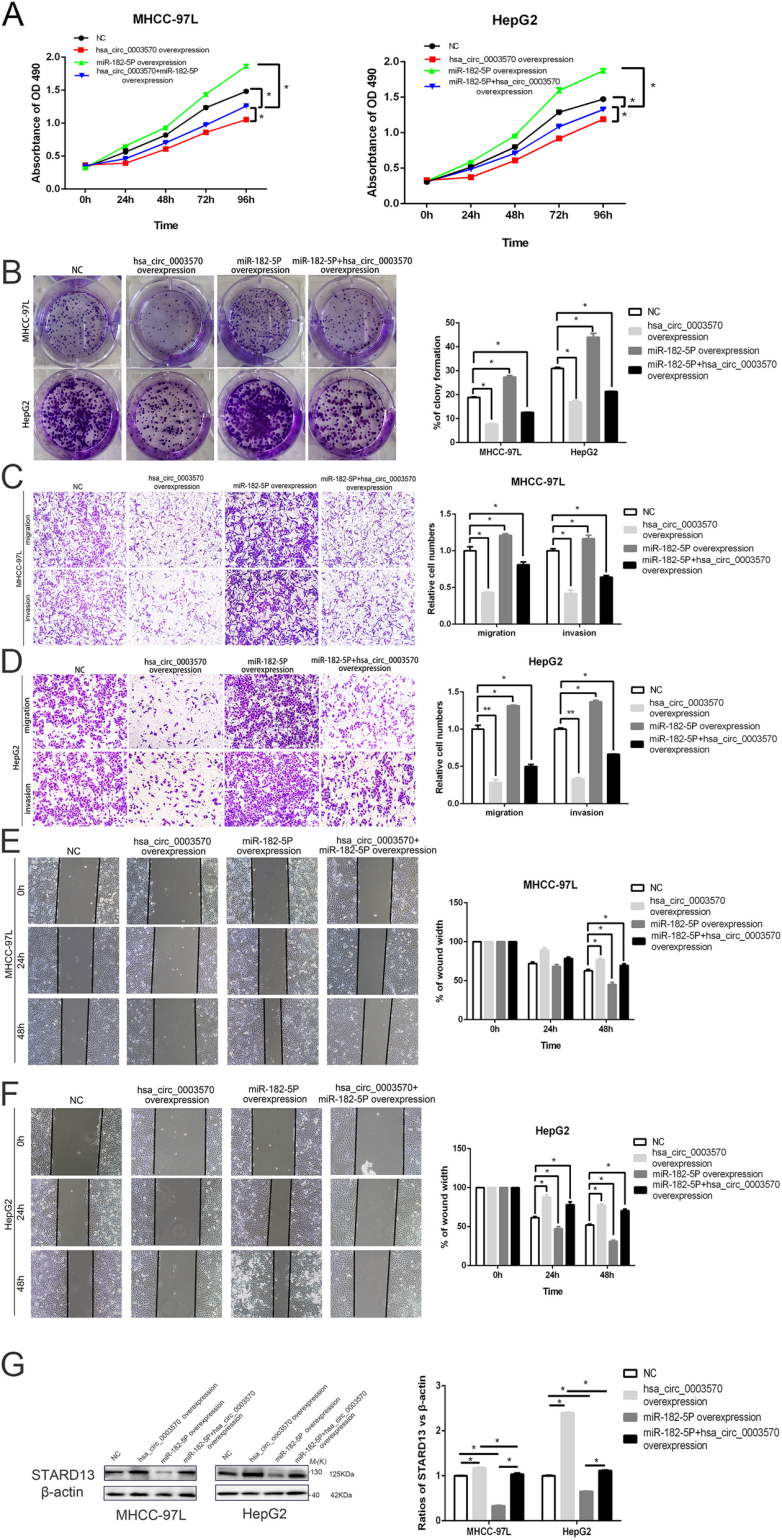
Circ_0003570 suppresses HCC proliferation, migration and invasion via the miR-182-5p/STARD13 axis. **A** The results of MTT assay showed that miR-182-5p mimics restored the inhibition of LV-circ_0003570 on cell proliferation over time. HCC cells grew faster in LV-circ_0003570 and miR-182-5p mimics co‐transfection group than LV-circ_0003570 group. *P < 0.05. **B** Colony formation assay showed that the proliferative capability of HCC cells was increased by miR-182-5p mimics compared with LV-circ_0003570 group. *P < 0.05. **C**, **D** The migration and invasion capacity restrained by upregulation of circ_0003570 could be reversed by miR‐182‐5p mimics. Cells migrated and invaded were increased in LV-circ_0003570 and miR-182-5p mimics co‐transfection group. *P < 0.05; **P < 0.01. **E**, **F** In wound healing assay, miR‐182‐5p mimics reversed the inhibitory role of LV-circ_0003570 on migration in HepG2 and MHCC-97L cells. *P < 0.05. **G** In protein level, the upregulation of STARD13 regulated by LV-circ_0003570 could be rescued by miR‐182‐5p mimics. One-way ANOVA was performed to compare the difference between two groups

Results mentioned above proved that circ_0003570 directly binds to miR-182-5p, we thus investigated circ_0003570 exerts a tumor-suppressing effect on HCC cells via sponging miR-182-5p. MTT assay was used to determine the proliferative capacity of circ_0003570 HCC cells in response to miR-182-5p mimics transfection. Interestingly, the proliferation ability of HCC cells co-transfected with miR-182-5p mimics and circ_0003570 was enhanced compared with that of HCC cells transfected only with LV-circ_0003570, indicating that overexpression of miR-182-5p could partly abolish the inhibition of proliferation induced by circ_0003570 (Fig. 4A). In concordance with the MTT results, the colony formation results showed that the inhibitory effect of circ_0003570 on HCC cell colony formation capacity could be partially abolished by miR-182-5p mimics (Fig. 4B). Similarly, transwell assay showed that miR-182-5p mimics apparently promoted the migration and invasion ability of HCC cells compared with circ_0003570 group (Fig. 4C-D). In wound healing assay, up-regulation of miR-182-5p partly attenuated the circ_0003570 mediated suppression of migration in HepG2 and MHCC‐97L cells (Fig. 4E-F). Meanwhile, western blotting indicated that the expression of STARD13 protein was markedly increased when circ_0003570 was upregulated, whereas the promotion of LV-circ_0003570 on STARD13 could be partly restored by miR-182-5p mimics (Fig. 4G). In conclusion, these results demonstrated that circ_0003570 regulates HCC cell growth and metastasis by suppressing miR-182-5p/STARD13 axis.

### Overexpression of circ_0003570 represses tumor growth and lung metastasis in vivo

We next proved the oncogenic effect of circ_0003570 in vivo via subcutaneously injecting HepG2 cells stably transfected with vector to establish xenograft model and injecting intravenously to form evaluate lung metastasis. The transfection efficiency of.

**Fig. 5:**
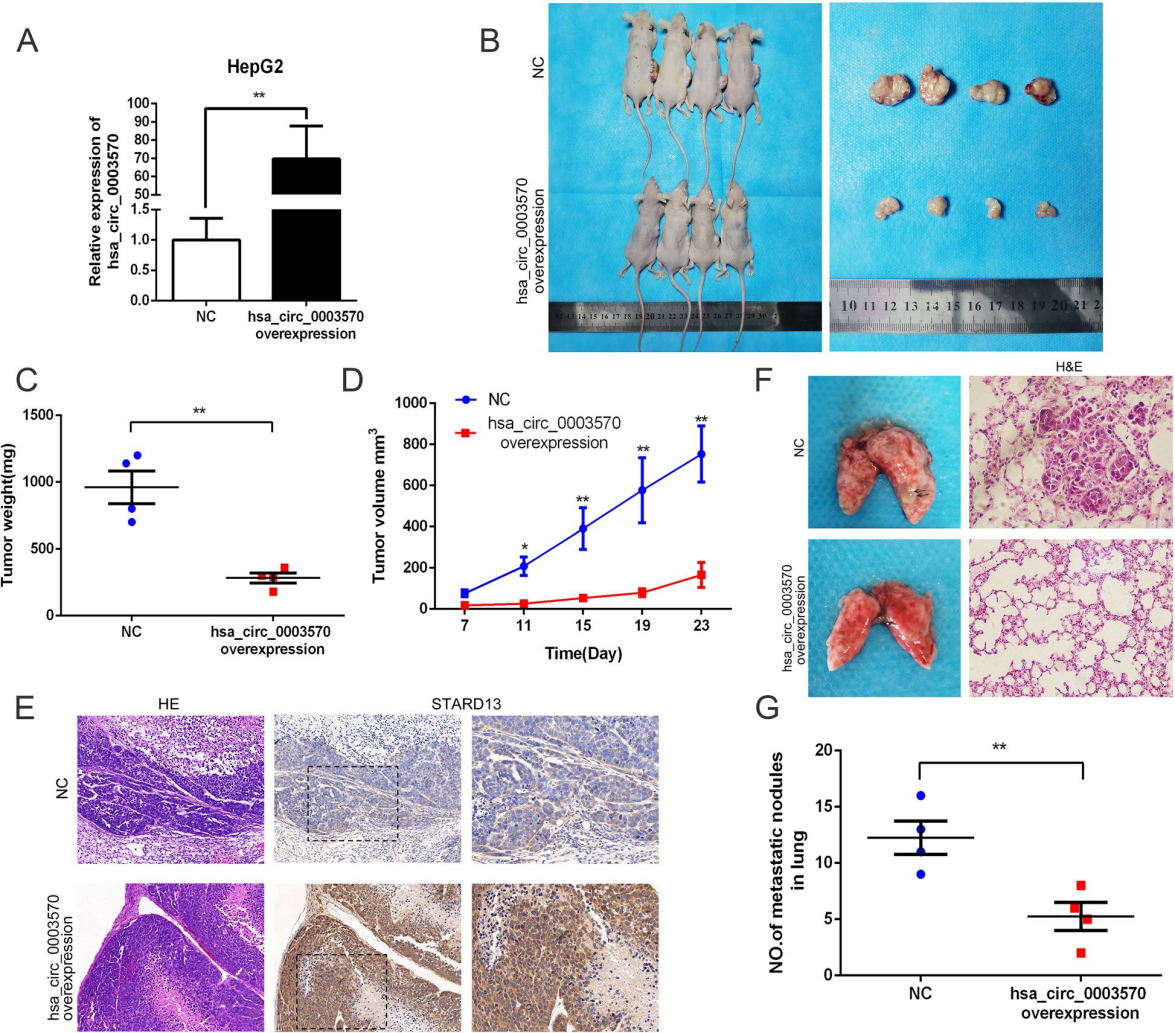
Overexpression of circ_0003570 represses tumor growth and lung metastasis in vivo. **A** The transfection efficiency of HepG2 cells stably transfected with LV-circ_0003570 was detected by qRT‐PCR, and the result showed that the expression of circ_0003570 was evidently overexpressed. **P < 0.01. **B** Representative images of nude mice modles and formed tumors that were subcutaneously injected with LV- circ_0003570 and NC cells. **C**, **D** Effect of circ_0003570 overexpression on HCC growth in vivo according to the tumor growth curve and tumor weight. *P < 0.05; **P < 0.01. **E** Representative images of HE and IHC staining patterns for STARD13 in tumor xenografts of nude mice (Left:10 × ; Right: 40 ×). **F**, **G** Representative images of pulmonary metastatic models and HE staining of metastatic nodules in the lungs (20 ×). Black arrows indicate metastatic nodules. **P < 0.01.Student’s t-test was performed to compare the difference between two groups

HepG2 cells was firstly confirmed by qRT‐PCR (Fig. 5A). Obviously, circ_0003570 dramatically hindered tumor growth compared with the vector group (Fig. 5B). The tumor volume and weight were significantly suppressed compared to those of the NC group (Fig. 5C-D). In addition, the expression of STARD13 was detected in xenograft tumors by IHC. The results showed that STARD13 was upregulated in xenograft tumors compared with the NC group (Fig. 5E). For pulmonary nude mouse model, there were obviously a larger number of lung metastatic nodules in NC group than that of the circ-overexpressed group. Besides, H&E staining of lung sections revealed that circ_0003570 upregulation decreased the number and size of pulmonary metastases (Fig. 5F-G). Briefly, circ_0003570 overexpression distinctly represses HCC tumor and pulmonary metastasis in vivo.

**Fig. 6:**
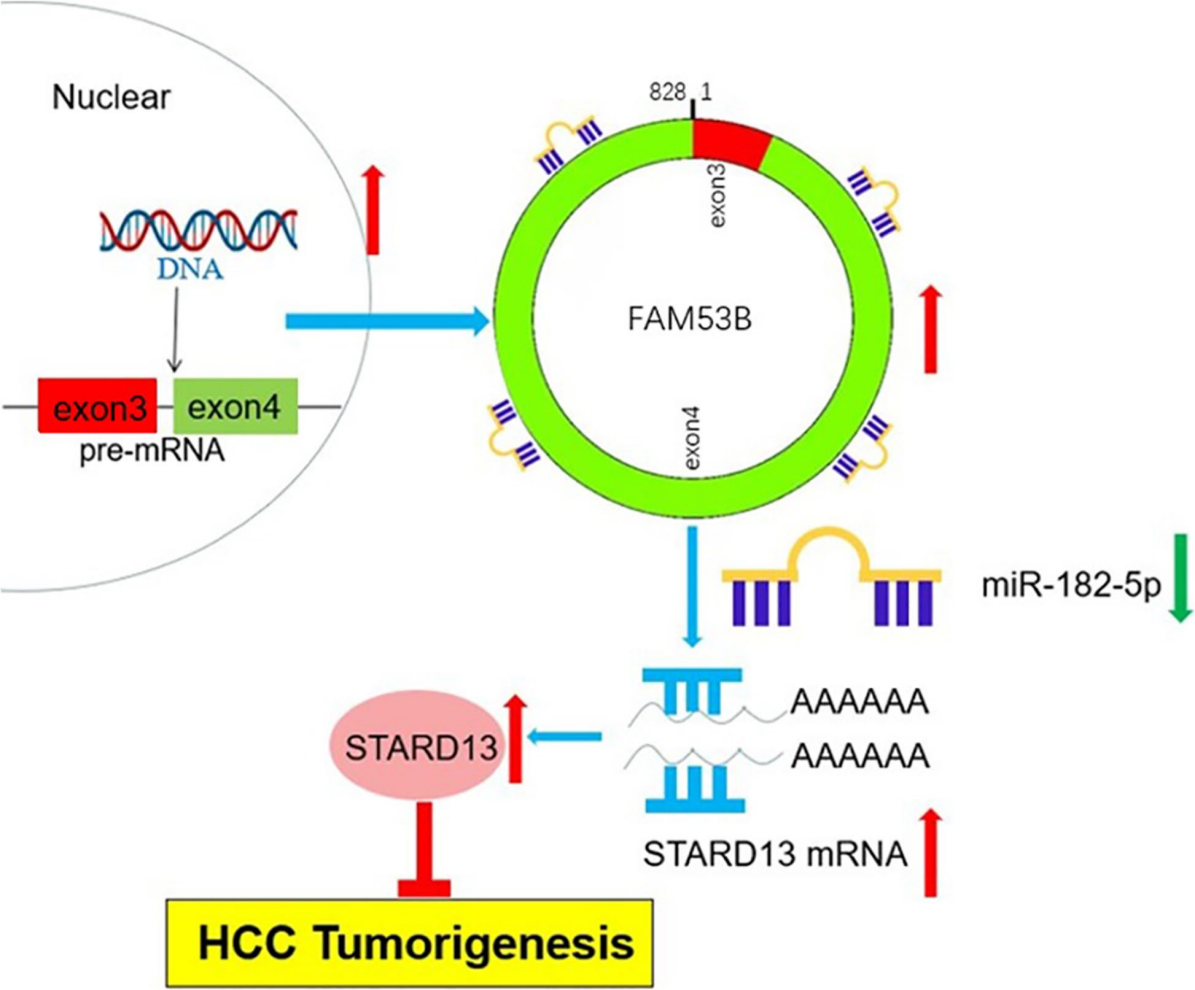
Schematic diagram depicting the biological function of circ_0003570 on hepatocellular carcinoma development in this research. Circ_0003570 suppressed HCC tumorigenesis by competitively binding miR-182-5p and modulating STARD13 expression

All these results suggested that circ_0003570 suppresses the progression of HCC potentially by competitively binding miR-182-5p and regulating the repression of target gene STARD13 (Fig. 6).

## Discussion

Recently, emerging studies have discovered that circRNAs participate in the progression and tumorigenesis of various human tumors, and might have potential to serve as prognostic biomarkers [18–20]. Since circ_0003570 is a newly discovered and previously rarely reported circRNA, its biological significance remains largely unknown. Tian et al. [21] found that circ_0003570 played a cancer-promoting role in infantile hemangioma (IH), and acted as the ceRNA of hsa-miR-138-5p and upregulated the expression of RGS5 to promote IH cell proliferation and inhibit IH cell apoptosis. Recently, a clinicopathological research [14] found that circ_0003570 expression levels were significantly lower in HCC tissues and HCC cell lines than that in adjacent normal tissues and human normal hepatic LO2 cell line. Meanwhile, the backsplicing sites and the circular structure of circ_0003570 were validated by qRT-PCR and Sanger sequencing. Moreover, its expression level was associated with HCC clinicopathological characteristics, such as tumor size, differentiation, lymph node metastasis, microvascular invasion, and the serum AFP level, suggesting that circ_0003570 might be a helpful prognostic biomarker of HCC. However, the molecular mechanisms of circ_0003570 in HCC have not been intensively studied yet. Hence, the present study aimed to thoroughly elaborated the its biological effects on HCC at the molecular level.

To further investigate the mechanism, the circ_0003570 function was explored using siRNA mediated knockdown experiments and overexpressed lentiviral vector mediated overexpression experoments. Upregulation or knockdown of circ_0003570 significantly affected HCC cell proliferation, migration and invasion in vitro. Meanwhile, xenograft tumor model revealed that overexpression of circ_0003570 markedly hindered tumor growth and lung metastasis in vivo. Collectively, our results demonstrated that circ_0003570 exerts tumor suppressor effects in HCC tumorigenesis and progression.

Although we have proved that circ_0003570 functions as a tumor suppressor in HCC, the potential regulatory mechanism by which circ_0003570 participates in tumor development remains to be elucidated. Studies have shown that circRNA could act as miRNA sponge, and regulate the certain circRNA‐miRNA‐mRNA axes involved in tumor pathogenesis, naming ceRNA [22, 23]. In other words, circRNA could function as a ceRNA to absorb microRNA competitively and indirectly improve the protein expression of target genes of miRNAs [24, 25]. In the present study, we aimed to illustrate the potential mechanisms by which circ_0003570 acts as a ceRNA in the development of HCC. We speculated that circ_0003570 could mediate HCC cell progression by interacting with miR-182-5p, and through qRT-PCR and dual luciferase reporter assay, we discovered that circ_0003570 directly binds to miR-182-5p and there existed an interactive suppression between them.

Previous studies have demonstrated that STARD13 acts as a crucial tumor suppressor gene in cancer, such as prostate cancer [26], colon cancer [27], breast cancer [28], and lung cancer [29]. A recent study [16] indicated that STARD13 could enhance 5-FU sensitivity by suppressing cancer stemness in hepatocellular carcinoma cells via attenuating YAP transcriptional activity. Another study [17] proved that STARD13 3′-UTR promotes cellular apoptosis by acting as a ceRNA for Fas in HCC cells. Simultaneously, it revealed that STARD13 could be a potential miRNA sponge, and its related ceRNA networks might be a rational therapeutic or diagnostic chance for HCC progression. In the present study, bioinformatics analysis revealed that STARD13 was a target gene of miR-182-5p. Our results demonstrated that circ_0003570 could sequester endogenous miR-182-5p to regulate STARD13 expression. On the other hand, we found that STARD13 could inhibit proliferation and metastasis of hepatocellular carcinoma.These results showed that circ_0003570 functions as an endogenous “sponge” by competitively binding miR-182-5p and thus abolishing miRNA-mediated repression of STARD13.

## Conclusions

In summary, the present study revealed that circ_0003570 was a novel tumor suppressor which inhibited HCC tumorigenesis and progression through competitively sponging miR-182-5p. Our research provided a deep understanding of the biological role of circ_0003570 and indicated that circ_0003570 might be a molecular target for clinical diagnosis and treatment of HCC.

## Supplementary Information

**Additional file 1: Figure S1.** The vector map of (A) wt-circ_0003570-luc, (B) mut-circ_0003570-luc; (C) wt-STARD13-luc, and (D) mut-STARD13-luc.** Figure S2.** Knockdown of circ_0003570 promotes proliferation, migration and invasion of MHCC-97L cells in vitro. (A) MTT assays and (B) colony formation assays showing that downregulation of circ_0003570 promoted the proliferation of MHCC-97L cells, *P < 0.05. (C) Transwell assays showed circ_0003570 knockdown reduced the migration and invasion of MHCC-97L cells. *P < 0.05. (D) Representative images of wound healing assays after circ_0003570 silencing. Student’s t-test was performed to compare the difference between two groups. *P < 0.05.** Figure S3.** Flow cytometric analysis. (A) Annexin-V and PI staining. For apoptosis analyses, cells were stained with propidium iodide (PI) and FITC-Annexin V, followed by flow cytometric analysis. (B) Cell cycle analysis of PI stained cells. For cell cycle analyses, cells were stained with propidium iodide (PI) and analyzed on flow cytometer. FACS analysis showed downregulation or upregulation of circ_0003570 did not affect apoptosis and cell cycle progression in HCC cells.** Figure S4**. MiR‐182‐5p promotes proliferation, migration and invasion of HCC cells. (A) qRT-PCR analysis of miR-182-5p expression following transfected MHCC-97L and HepG2 cells with miR-182-5p mimics or inhibitor. *P < 0.05; **P < 0.01. (B, C) The results of MTT assays revealed that miR-182-5p mimics promoted HCC cell proliferation while miR-182-5p inhibitor hindered cell proliferation. *P < 0.05; **P < 0.01. (D) In clone formation assay, miR-182-5p mimics promoted HCC cell proliferation while miR-182-5p inhibitor hindered cell proliferation. *P < 0.05. (E,F) In transwell assay, the migration and invasion capacity of HCC cells was induced by miR-182‐5p mimics and apparently reduced by miR‐182‐5p inhibitor. *P < 0.05, **P < 0.01. (G, H) The results of wound healing assay showed that HCC cells moved faster in miR-182-5p mimics group while miR-182-5p inhibitor obviously reduced the mobility of HCC cells. *P < 0.05.Student’s t-test was performed to compare the difference between two groups.** Figure S5.** STARD13 inhibits proliferation, migration and invasion of HCC cells. (A) Western blotting analysis of STARD13 expression following transfected HCC cells with STARD13 siRNA or overexpression plasmid. *P < 0.05; **P < 0.01. (B) The results of MTT assays revealed that STARD13 knockdown promoted HCC cell proliferation while STARD13 overexpression hindered cell proliferation. *P < 0.05; **P < 0.01. (C) In clone formation assay, STARD13 knockdown promoted HCC cell proliferation while STARD13 upregulation hindered cell proliferation. *P < 0.05. (D) In transwell assay, the migration and invasion capacity of HCC cells was induced by STARD13 siRNA and apparently reduced by STARD13 plasmid. *P < 0.05, **P < 0.01. (E) The results of wound healing assay showed that HCC cells moved faster in STARD13 downregulation group while STARD13 upregulation obviously reduced the mobility of HCC cells. *P < 0.05.Student’s t-test was performed to compare the difference between two groups

## Data Availability

The data are available from the corresponding author upon reasonable request.
